# Reducing pre-operative delays in urgent cardiac surgery: evidence for waiting-time effect on post-operative length of stay

**DOI:** 10.1186/s13019-026-04551-6

**Published:** 2026-08-08

**Authors:** Sarah Raut, Alan Race, David Yates, Lee Ingle

**Affiliations:** 1 Hull University Teaching Hospitals NHS Trust, Hull, UK; 2 York and Scarborough Teaching Hospitals NHS Foundation Trust, York, UK; 3 North Yorkshire Academic Alliance of Perioperative Medicine, York, UK; 4 School of Sport, Exercise & Rehabilitation Science, Faculty of Health Sciences, University of Hull, Hull, UK

**Keywords:** Cardiac surgery, Pre-operative length of stay, Urgent cardiac surgery, Urgent cardiac surgery outcome, Post-operative outcome

## Abstract

**Background:**

According to the National Institute of Cardiovascular Outcomes Research (NICOR) report in 2022, the mean waiting time for urgent coronary artery bypass graft (CABG) surgery in the United Kingdom is 10 days, rather than the recommended 7 days. We conducted a service evaluation of the acute cardiac pathway in our hospital.

**Methods:**

All patients who received cardiac surgery in 2022 were considered. 611 patients had cardiac surgery; 585 had elective or acute cardiac surgery; 580 had complete data for analysis. Data were reported in counts and percentages. We determined the associations between pre-operative length of stay and post-operative outcomes using negative binomial analysis. Fine-Gray model was used to address survival bias.

**Results:**

Median pre-operative LOS for urgent patients referred directly to CHH was 12 days (IQR 8.0-16.5, *p* < 0.001). Each additional pre-operative day were associated with an increase of stay (LOS) by 3.2%. Sensitivity analysis on the pre-operative LOS rate ratio shows that this effect is purely confined to the urgent population; RR 1.030, *p* < 0.001 and absent in elective patients; RR 0.976, p 0.3966. The effect is enhanced in patients presenting to Castle Hill Hospital (CHH) directly as we were able to capture the full duration of their in-hospital LOS; RR 1.035, *p* < 0.001. Other factors that impact post-operative LOS were presenting ejection fraction (EF), type of surgery and age.

**Conclusion:**

Pre-operative LOS was independently associated with prolonged post-operative stay. This effect is confined to the urgent population supporting a service improvement focus on reducing in-hospital delays for the urgent pathway.

## Introduction

Surgical waiting lists can be a challenge to manage as demand often exceeds supply. To enable timely delivery of care to the masses, care is prioritised according to clinical urgency. The impact of waiting times on patient outcomes is usually evaluated via retrospective or observational analysis as it is unethical to intentionally withhold evidence-based, clinically effective treatments. In cardiac surgery, prolonged waiting times have been associated with increased risk of emergency admission [1] and reduced physical functioning [2]. 

Between 2000 and 2010, the waiting time for elective coronary artery bypass graft surgery (CABG) fell from 220 days to 50 days [1]. Unfortunately, this is no longer the case as CABG waiting times have increased between 21 and 94% since the Covid-19 pandemic across the United Kingdom (UK) [3]. The pandemic not only delayed the elective surgical pathway for CABG, but also prolonged the in-patient waiting time for acute surgery. This is partly due to the diversion of resources to prioritise care for COVID-19. This in turn led to reduced hospital bed capacity and affected bed-flow. In addition, due to the extra precautions required for COVID-19, productivity is impacted. NICOR recommended a target of 75% urgent CABG surgeries be performed within 7 days of angiography in the UK [3, 4] This target time allows for anti-platelets and anticoagulation therapy to diminish. Unfortunately, only 3 hospitals in the UK managed to achieve this target [3]. The average waiting time for a CABG is 10 days in the UK [3]. Functional decline could occur within 24 h of hospital admission [5] and muscle deconditioning in as little as 5 days [6]. Hospital associated deconditioning impacts not just physical but also cognitive function [5]. 

In the North Eastern region of England, Castle Hill Hospital (CHH) situated in Kingston-upon-Hull, was one of the many hospitals in the UK that were unable to meet the 7 days target for urgent CABG. The hospital experienced a reduction in cardiac surgery of nearly 50% in 2022, making it the hospital with the second most disrupted cardiac surgical activity in the UK [7]. As a tertiary hospital, CHH receives direct admissions as well as referrals from District General Hospital (DGH) in its catchment area. Patients are initially admitted under the cardiology team for review and investigations. Patients would either undergo an intervention, or their case be discussed at the multidisciplinary team meeting (MDT). If, after this process, patients are to be listed for surgery, they will be referred to the cardiac surgeons, assessed by the surgical team and prepared for surgery. Patients are often listed according to clinical urgency. The day of surgery could also be impacted by factors such as theatre and intensive care beds availability. This means that there could be unexpected changes in surgical schedules.

The extended waiting time and the negative implications of being hospitalised led us to consider the potential of acute prehabilitation. Prehabilitation typically involves a combination of exercise training, nutritional support, and psychological interventions; as recommended by Enhanced Recovery After Cardiac Surgery (ERAS-Cardiac). Exercise training, especially inspiratory muscle training, has shown positive outcomes in improving respiratory function and reducing post-operative pulmonary complications (PPCs) [8, 9]. Despite the potential benefits, the implementation of prehabilitation in cardiac surgery is limited in the UK. A recent survey indicated that only 13% of UK cardiac surgery centres offered any form of prehabilitation, and this was primarily available to patients on the elective pathway [10]. With the challenges faced in implementing elective prehabilitation, acute patients have been somewhat ignored.

Consequently, the aim of our study was to conduct a service evaluation of an existing pathway at CHH in Kingston-upon-Hull to understand the duration of time a patient spends in the pathway, reason(s) they are waiting, identify scheduling challenges during the waiting time, and calculate the associations between waiting times and patient outcomes. Understanding these challenges will help to answer some of the questions regarding the feasibility of a prehabilitation pathway within this patient cohort.

## Method

Institutional approval for this service evaluation was granted by the hospital’s Audit and Clinical Governance Committee. Formal ethical approval and patient consent is not required for this project as per Health Research Authority decision tool. All patients admitted to CHH for cardiac surgery automatically have their clinical information collected by a database manager. This database is used to provide information as required for national audit, service evaluation or research purposes. 611 patients had cardiac surgery in CHH in 2022. As we were mainly interested in those having elective or acute surgery, 585 patients were eligible. However, due to missing values, only 580 could be included in the statistical analysis. Information was requested from the database manager with some supplementation from their electronic patient record (Lorenzo).

We have defined the urgency of surgery according to the manual of perioperative care in adult cardiac surgery. Elective patients were those with stable cardiac function; as such, their surgery could be deferred [11], and subsequent admission is planned and booked in advance. Urgent patients were unplanned admissions that required definitive treatment during their current admission, prior to discharge [11]. Emergent salvage surgery is when a patient is undergoing CPR en-route to the operating theatre prior to anaesthesia induction [11]. Emergency surgeries are those performed if the patient’s clinical status includes any ischaemic or mechanical dysfunction [11]. Ischaemic dysfunction could include ongoing ischaemia despite maximal medical therapy, acute evolving myocardial infarction (MI) in the 24 h before surgery, or pulmonary oedema requiring endotracheal intubation and ventilation [11]. 

Data is reported in counts and percentages. Continuous data were presented as median and interquartile range (IQR). Categorical data were presented as counts and percentages. Wilcoxon rank-sum test were performed on continuous data and Fisher exact test were performed on categorical data. To control family-wise error rate, p-values were Holm corrected for multiple comparisons. We performed negative binomial regression and sensitivity analysis on pre-operative LoS to post-op LoS. Post-operative LOS exhibited marked over dispersion (variance-to-mean ratio 17.8), precluding poisson regression. As such, a negative binomial model was fitted (dispersion parameter theta = 2.84). Analysis was undertaken using R statistical software (V4.4.0; R Core Team 2024).

## Results

611 patients had cardiac surgery in CHH in 2022. 322 (52.7%) patients were admitted electively. 263 (43.0%) patients were classified as urgent. 26 (4.3%) patients had emergency/salvage surgery. We analysed a total 585 acute and elective patients for the purposes of our study. Due to missing values on model variables, 5 patients were excluded from negative binomial regression analysis; this leaves 580 eligible patients.

**Table 1:** Demographics according to pathway

	Elective (E)	Urgent CHH (U-C)	Urgent DGH (U-D)	E vs. U-C *p*-value	E vs. U-D *p*-value	U-C vs. U-D *p*-value
n	322	95	168			
**Demographics**						
Age, years, median (IQR)	70.0 (62.0–75.0)	65.0 (60.0–73.0)	68.0 (60–73.2)	0.030	0.129	0.384
Male	223 (69.3%)	71 (74.7%)	126 (75.0%)	0.740	0.622	1.000
Female	99 (30.7%)	24 (25.3%)	42 (25.0%)			
**Ejection Fraction (EF)**						
EF: Good	263 (81.7%)	59 (62.1%)	109 (64.9%)	< 0.001	< 0.001	0.382
EF: Fair	50 (15.5%)	25 (26.3%)	48 (28.6%)			
EF: Poor	9 (2.8%)	11 (11.6%)	11 (6.5%)			
**Operation**						
CABG only	145 (45.0%)	62 (65.3%)	102 (60.7%)	0.013	< 0.001	0.364
Valve Only	113 (35.1%)	19 (20.0%)	30 (17.9%)			
CABG + valve	24 (7.5%)	6 (6.3%)	21 (12.5%)			
Major aortic	33 (10.2%)	5 (5.3%)	13 (7.7%)			
Other	7 (2.2%)	3 (3.2%)	2 (1.2%)			
**Length of stay (LOS)**						
Pre-op LOS, days, median (IQR)	1.0 (1.0–1.0)	12.0 (8.0–16.5)	5.0 (2.0–9.2)	< 0.001	< 0.001	< 0.001
Post-op LOS, days, median (IQR)	7.0 (5.0–10.0)	7.0 (5.0–12.5)	7.0 (5.0–10.0)	1.000	1.000	1.000
ICU hours, median (IQR)	45.0 (25.0–79.8)	51.0 (27.0–94.0)	48.0 (25.5–78.6)	0.279	0.598	0.598
**Outcomes**						
In-hospital death	6 (1.9%)	5 (5.3%)	6 (3.6%)	-	-	-
Post-op AF/flutter	150 (46.6%)	44 (46.3%)	68 (40.7%)	1.000	0.749	0.872
Reintubation	13 (4.0%)	8 (8.4%)	5 (3.0%)	0.219	0.623	0.219

IQR, interquartile range; CABG, Coronary Artery Bypass Graft; ICU, Intensive Care Unit; AF, Atrial Fibrillation; E vs. U-C, Elective vs. Urgent CHH pairwise p-value; E vs. U-D, Elective vs. Urgent DGH pairwise p-value; U-C vs. U-D, Urgent CHH vs. Urgent DGH pair wise p- value

Table 1 shows patient demographics according to their pathways. We have separated patients into three categories; electives (both from CHH and DGH), urgent CHH and urgent DGH. All elective patients are admitted the day before surgery to CHH. Urgent patients from DGH stay in DGH for an unknown period of time for various reasons such as pre optimisation, investigations and awaiting a cardiac surgical bed to be available in CHH. Continuous data are presented as median and interquartile range. Categorical data are presented as counts and percentages. Pairwise p-values were performed comparing elective to urgent CHH, elective to Urgent DGH and urgent CHH to Urgent DGH.

Of the 585 patients, 322 were electives, 95 were direct urgent admission to CHH and 168 were admission via DGH. The DGH included York, Scarborough, Bridlington, Malton, Selby, Grimsby, Scunthorpe, and Goole hospital. The median age in the elective group was 70 years (62.0–75.0), urgent CHH 65 years (60.0–73.0), and urgent DGH 68 years old (60–73.2). The proportion of male patients were higher in all three categories; up to three times more male patients compared to female patients. Pairwise p-values shows that there is no statistical difference in age or gender distribution amongst the three groups.

There was higher proportion of poor ejection fraction (EF) on admission amongst urgent patients. 81.7% electives had good EF, 14.5% had fair EF and 2.8% had poor EF. 62.1%, 26.3% and 11.6% urgent patients presenting directly to CHH had good, fair and poor EF respectively. In the urgent group from DGH, 64.9% had good EF, 28.6% fair EF and 6.5% poor EF on initial presentation. Pairwise p-value indicates statistical significance (*p* < 0.001) between the elective and urgent group. However, when comparing the two urgent groups, p-value was 0.382.

The most common operation performed was CABG 45% elective, 65.3% urgent CHH and 60.7% urgent DGH. Second most common operation was valve surgery; 35.1% elective, 20% urgent CHH and 17.9% urgent CHH group. Aortic valve replacement (AVR) was the most common valve surgery followed by mitral valve surgery. AVR was performed 4.6 times more than mitral valve surgery. 7.5% elective, 6.3% urgent CHH and 12.5% urgent DGH had both CABG and valve surgery. Major aortic surgery were surgery involving the root, ascending, arch of aorta with or without aortic valve surgery. 10.2% major aortic surgery were elective, 5.3% urgent CHH and 7.7% urgent DGH. Other surgery included procedures such as myxoma, pericardiectomy, sub-aortic membrane resection, repair of paravalvular leak. These procedures were relatively uncommon, making up 2.2% in the elective group, 3.2% urgent CHH and 1.2% urgent DGH. No difference was found when comparing the elective to urgent CHH (*p* = 0.013) and the two urgent groups (*p* = 0.364) in terms of surgical procedure. However, there was a difference between the elective and urgent DGH group (*p* < 0.001).

As expected, elective patients arrive the day before surgery and have their surgery the next day as planned. Urgent CHH patients had median stay of 12 days (IQR 8.0–16.5) compared to urgent DGH with median stay 5 days (IQR 2.0–9.2). Pairwise comparison of the three groups showed a difference in pre-operative LOS amongst the three group (*p* < 0.001). When comparing post operative LOS, the median for all three groups were the same; 7 days with IQR 5.0–10.0 days in the elective group, 5.0–12.5 days in urgent CHH and 5.0–10.0 days in the urgent DGH group. This is reflective of the most common operation being CABG and second most common being AVR. CABG and tissue AVR without post-operative complications are expected to stay in hospital 5–8 days after the surgery. There was no difference in post-operative length of stay detected amongst the three group (*p* = 1.000). The median ICU stay for elective patients was 45 h (IQR 25.0–79.8), urgent CHH was 51 h (IQR 27.0–94.0) and urgent DGH 48 h (25.5–78.6). There was no statistical difference in ICU stay amongst the three group.

Overall in-hospital mortality after surgery was significantly lower in the elective group (1.9%) compared to urgent CHH (5.3%) and urgent DGH (3.6%). There were no ICU readmission during the period of the study. The most common arrhythmia after cardiac surgery is atrial fibrillation (AF) and atrial flutter. The rate of post-operative AF/flutter was over 40% in all three groups; 46.6% in elective, 46.3% in urgent CHH and 40.7% in urgent DGH. No statistical difference in post-operative AF/flutter was detected amongst the three group. Rate of pulmonary re-intubation was 4% in elective, 8.4% in urgent CHH and 3% in urgent DGH patients. There was no difference in pulmonary re-intubation amongst the three groups. There were no post-operative stroke, renal filtration or ICU readmission during our period of study.

**Fig. 1:**
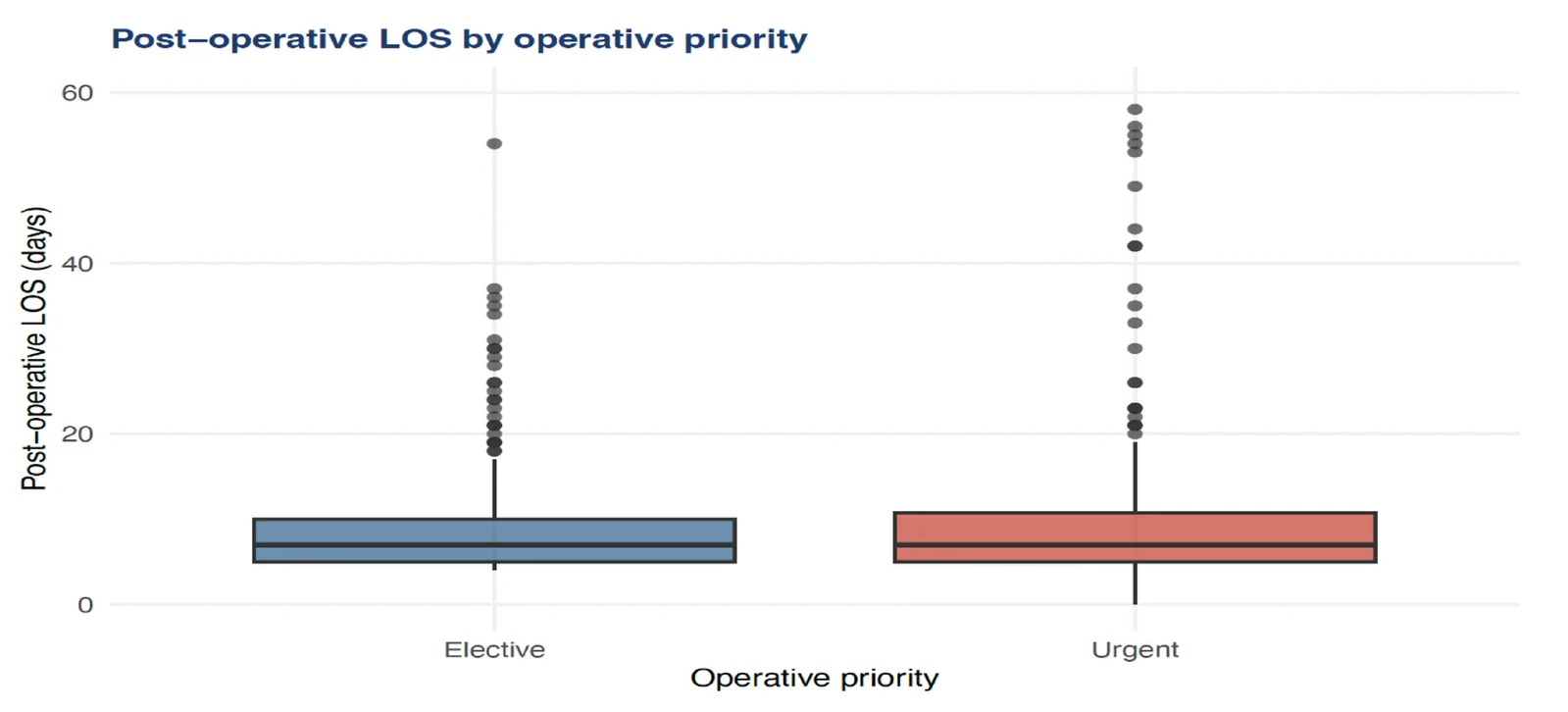
Post-operative LOS by operative priority

Figure 1 shows the post-operative LOS by operative priority. The box plot demonstrates that although the median post-operative LOS was similar for both groups, the urgent group had a wider and higher distribution of post-operative LOS. This reflects the greater physiological burden of the acute presentation. The box plot indicates that operative priority is both a confounder and effect modifier for post-operative LOS.

**Fig. 2:**
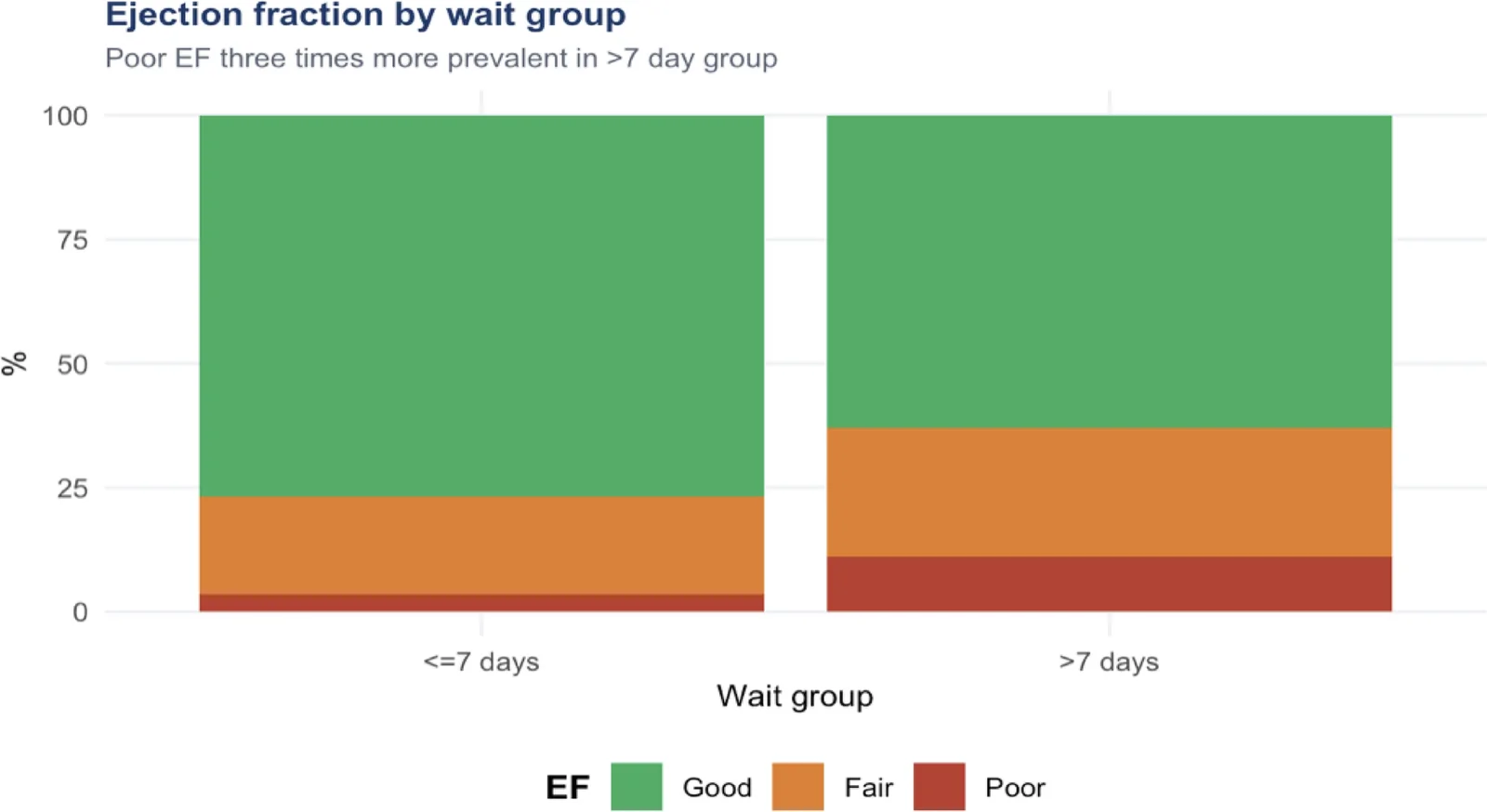
Ejection fraction by wait group

We considered the effect of EF on admission on waiting times. (Fig. 2) We divided the patients according to two groups, those that wait 7 days or less for their operation and those that waited more than 7 days for their operation. The 7 days allows time for investigations and anti-platelet therapy cessation as recommended by NICOR. We have used the 7 days threshold for descriptive context only. Patients who waited > 7 days for their operation had poorer ejection fraction on admission that patients who waited 7 days or less. Poor baseline ejection fraction was three times more prevalent in patients that waited > 7 days.

**Fig. 3:**
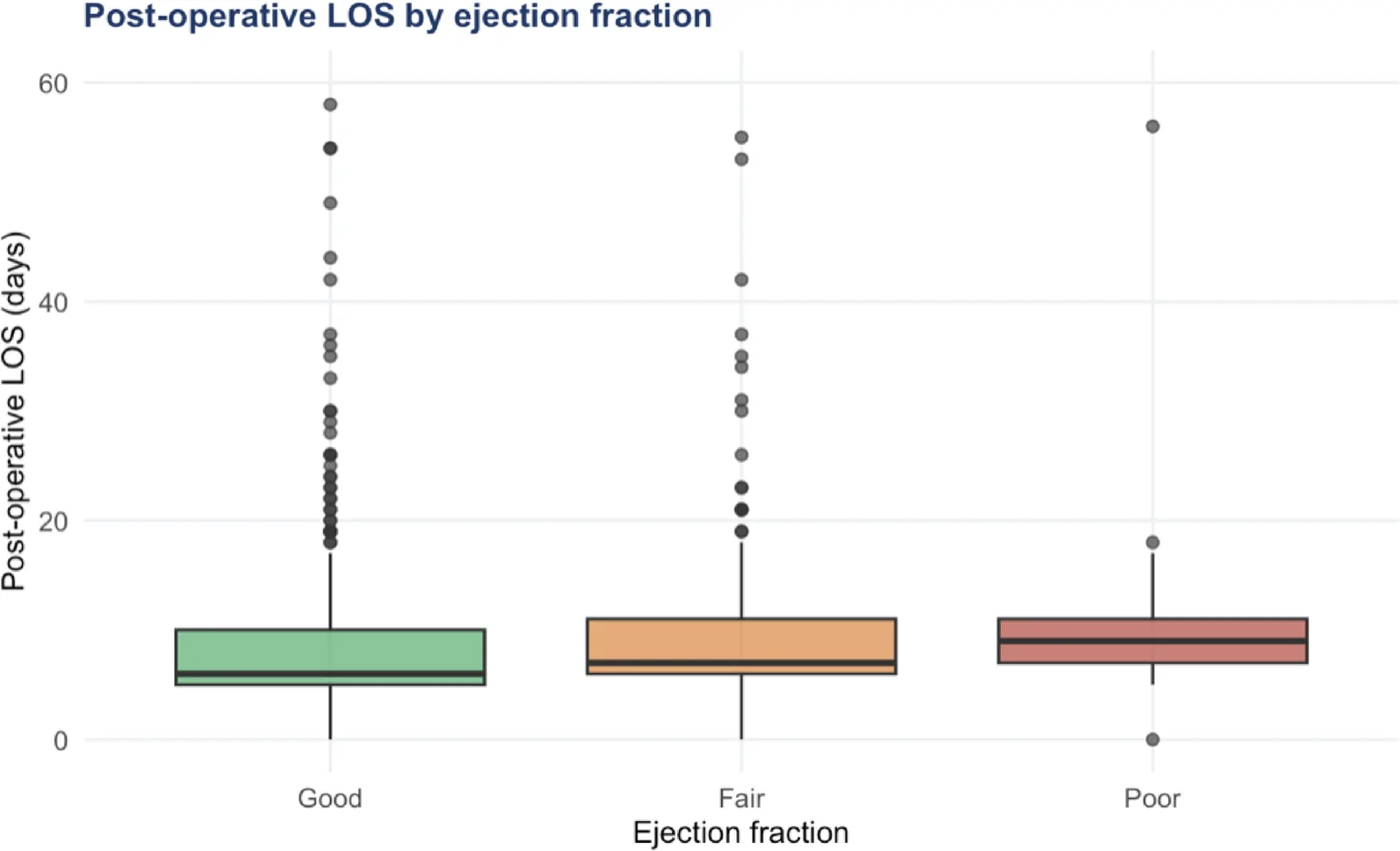
Post-operative LOS by ejection fraction

Unsurprisingly, poorer ejection fraction was associated with longer and more variable length of stay (Fig. 3). This meant

**Fig. 4:**
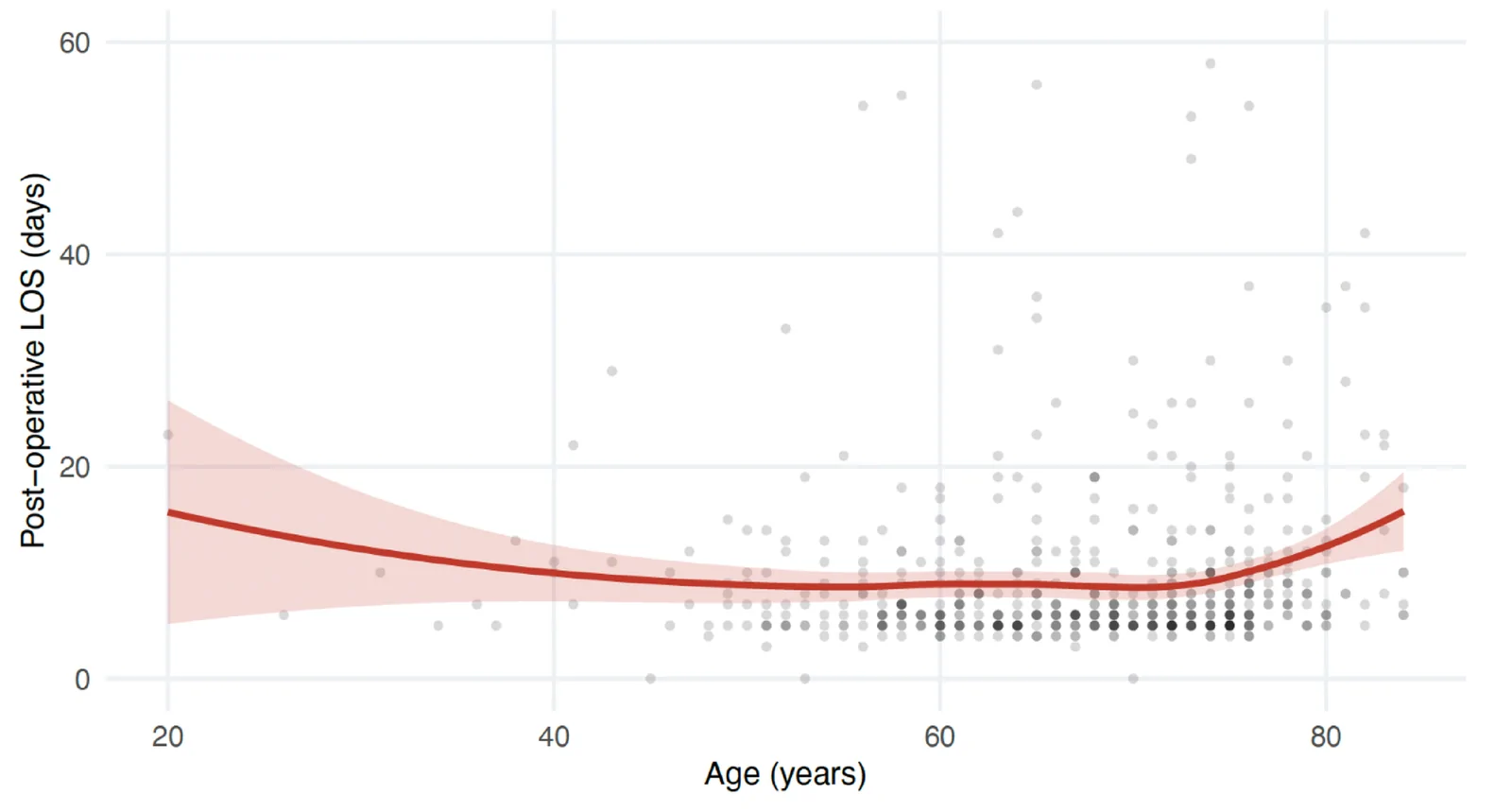
Post-operative LOS vs. age

that patients with poorer ejection fraction had longer total in-hospital stay compared to those with good ejection fraction.

We plotted the post-operative LOS to age (black dots), then drew a best line through this (red line). (Fig. 4) The relationship between post-operative LOS and age is non liner. The post-operative LOS was longer in younger patients, falls through the middle ages then rises again after 70 years old. Although, the number of younger patients (below 40 years old) are significantly lower, those that undergo cardiac surgery tend to have more complex operation. After 70 years old, the post-operative LOS increases likely due to the decline in their physiological reserve.

**Fig. 5:**
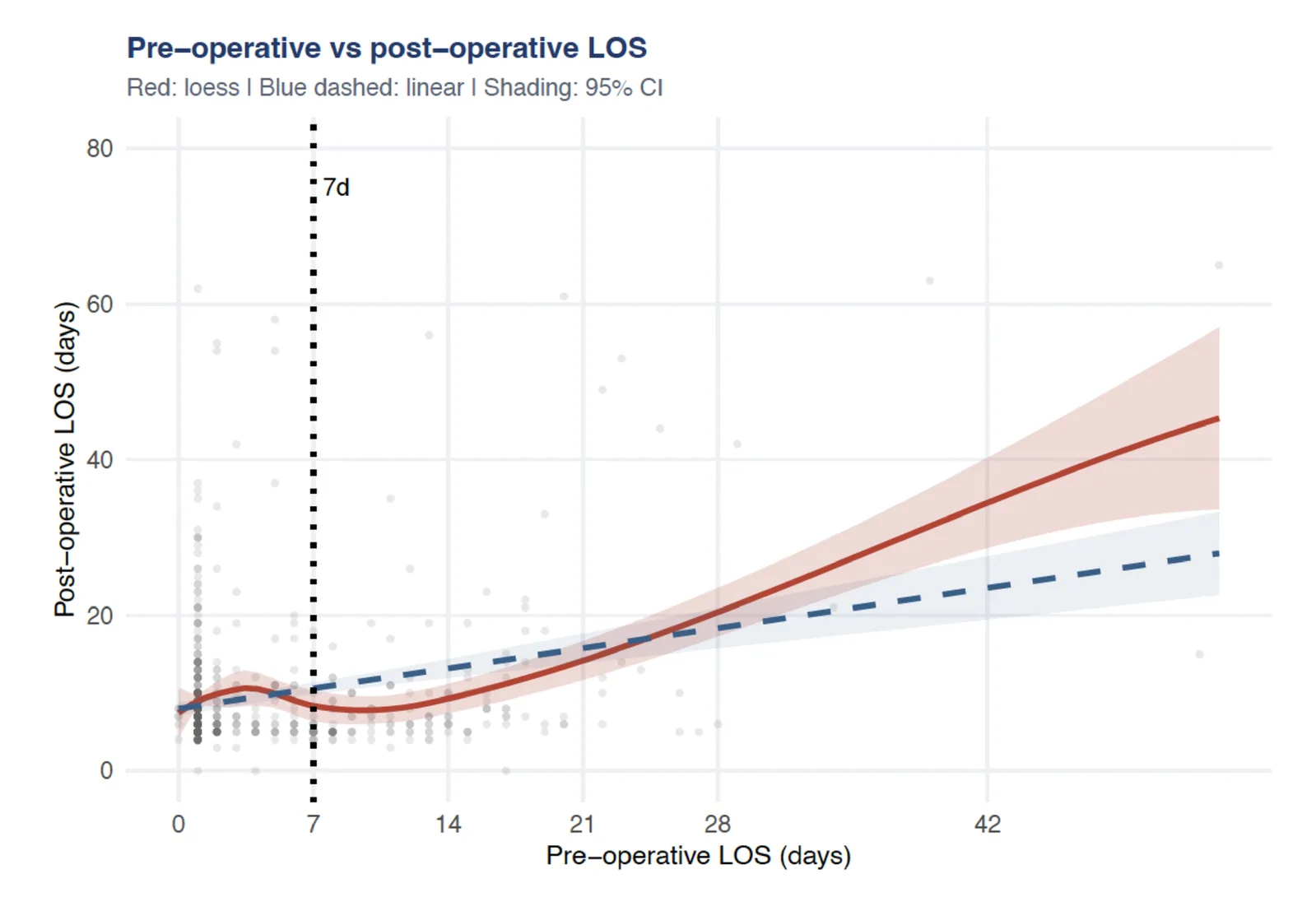
Pre-operative vs. Post-operative LOS

The red line in Fig. 5 is the locally estimated scatterplot smoothing (LOESS) line of pre-operative to post-operative LOS. The blue dashed line shows the linear Ordinary Least Square (OLS) which is the ‘best-fitting’ line which we used as reference line. Non-linearity was assessed by comparing the linear model with a restricted cubic spline specification (3 knots). The likelihood ratio test was non-significant (p-0.192, delta-AIC = 1.8), supporting the adequacy of the linear term. The shaded area represents 95% confidence interval range. We have also marked the 7 days as recommended by NICOR. This is used here for descriptive context only; all inferential analyses model pre-operative LOS as a continuous variable to preserve dose-relationship information. As seen above, the post-operative LOS does not vary significantly for pre-operative days of 7 days or less. Post-operative LOS hovers around the 8–9 days until after the 14th day of pre-operative LOS. The post-operative LOS relationship accelerates steeply beyond 14 days and around the 23 days pre-operative stay, it crosses the reference line and exceeds the OLS.

**Fig. 6:**
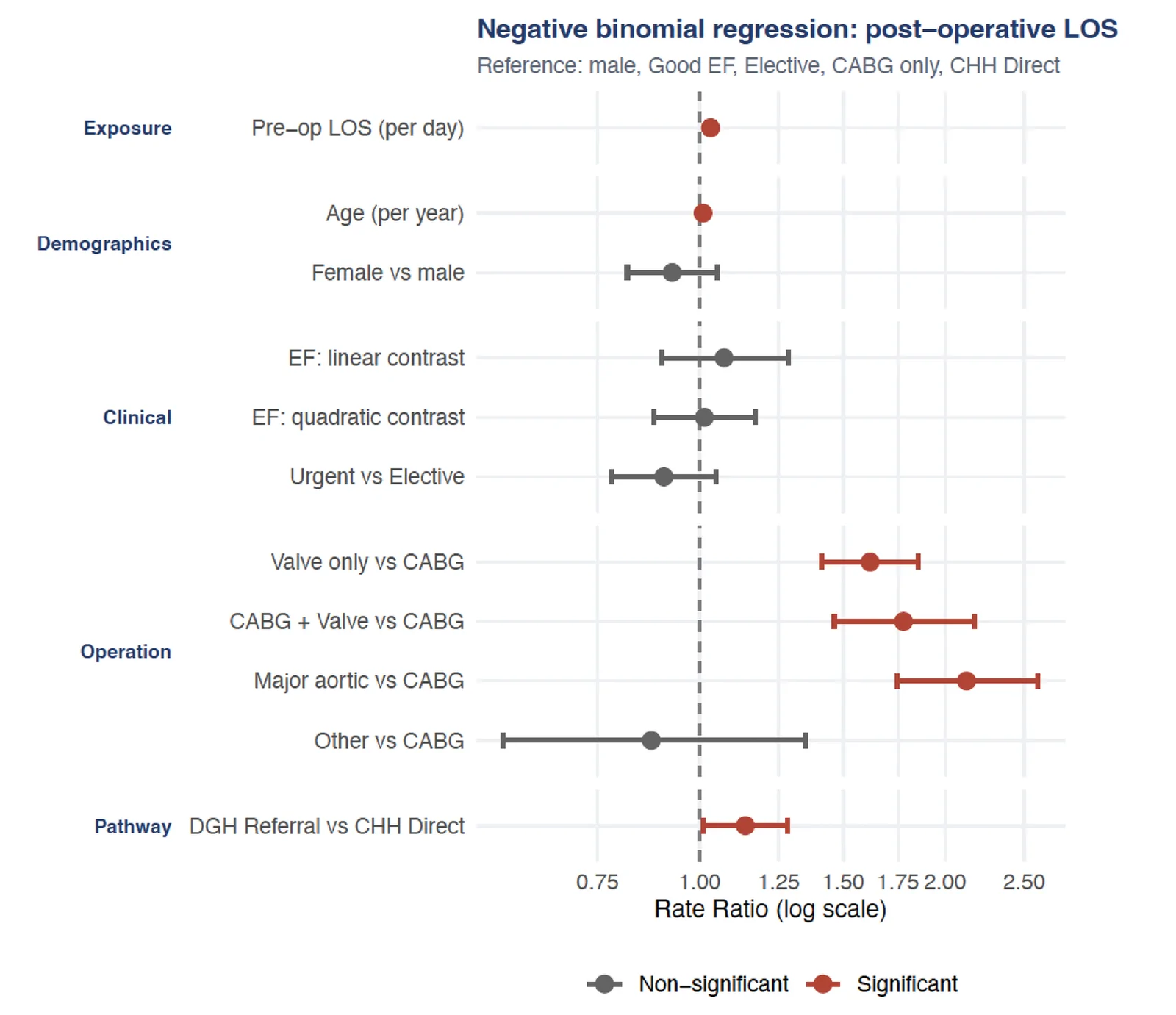
Forest plot representation of negative binomial regression

Table 2 shows the main results of our negative binomial regression model with fig. 6 forest plot representation of the results. We compared the post-operative LOS for each category. The primary adjusted pre-operative LOS RR was 1.032, CI 1.021–1.042, *P* < 0.001; each additional pre-operative day increases expected post-operative LOS by 3.2% after full confounder adjustment. There was no significant difference in the groups when comparing gender, ejection fraction or surgical emergency both with RR < 1 and p value > 0.05. We compared the different types of surgery to CABG which is the commonest adult cardiac operation performed. Valve (RR 1.618, 95% CI 1.413–1.855, *P* < 0.001), valve with CABG (RR 1.778, 95% CI 1.463–2.174, *p* < 0.001) and major aortic (RR 2.124, 95% CI 1.745–2.6, *p* < 0.001) had longer post-operative stay compared to CABG alone. Other surgery (*n* = 12 as per Table 1) category was not statistically significant likely due to the small number. The type of surgery mattered far more than pre-operative LOS in determining the length of post-operative stay. Patients referred from DGH stand 1.138 times longer in hospital after their surgery compared to those that present directly to CHH, 95% CI 1.011–1.281, *P* = 0.03.

**Table 2:** Negative binomial regression model analysis: post-operative LOS in days

Term	Rate Ratio	95% CI	p-value
Primary (adjusted)	1.032	1.021–1.042	<0.001
**Age (per year)**	1.01	1.004–1.016	0.001
**Female vs male**	0.926	0.815–1.052	0.234
**EF linear contrast**	1.072	0.9–1.285	0.438
**EF quadratic contrast**	1.014	0.88–1.169	0.85
**Urgent vs Elective**	0.904	0.781–1.047	0.174
**Valve only vs CABG**	1.618	1.413–1.855	<0.001
**CABG + Valve vs CABG**	1.778	1.463–2.174	<0.001
**Major aortic vs CABG**	2.124	1.745–2.6	<0.001
**Other vs CABG**	0.873	0.575–1.35	0.529
**DGH Referral vs CHH Direct**	1.138	1.011–1.281	0.03

LOS; Length of Stay, EF; Ejection fraction, CABG; coronary artery bypass graft, DGH; District General Hospital, CHH; Castle Hill Hospital

We performed subsequent analysis to identify impact of each bias on post-operative LOS. (Table 3) The unadjusted rate ratio was 1.024 (95% CI 1.016–1.033, *p* < 0.001). We adjusted for confounders for age, gender, ejection fraction, operative priority, type of surgery and admission source. Upon adjustment, every additional pre-operative day stay was associated with 3.2% longer post-operative stay (95% CI 1.021–1.042, *p* < 0.001). Adjustment increased the rate ratio from 1.024 to 1.032, consistent with negative confounding by severity; severity markers appears to suppress rather than inflate the pre-operative LOS association. Patients that stayed in hospital > 90 days were excluded (S1) to identify if outliers were driving the results. The RR for S1 was 1.029 (CI 1.02–1.039, *P* < 0.001). When the groups are divided into elective and urgent (S2 and S3), it shows that the effect of pre-operative LOS on Post-operative LOS is entirely confined to the urgent patient group (RR 0.976 vs. RR 1.03). Patients who came in electively would usually be admitted the day before surgery. The effect of longer pre-preoperative LOS was more pronounced when we considered only those that present directly to CHH. Each pre-operative day stay was associate with 3.5% longer post-surgery stay. The duration of pre-operative stay in this study was only recorded from the point the patient presents to CHH. This meant that those that present to DGH first will not have their full pre-operative stay recorded. S5 shows the result of subgroup sensitivity analysis to address survivor bias. When the 17 deaths were excluded, this shows RR 1.034 (95% CI 1.024–1.045, *p* < 0.001).

**Table 3:** Sensitivity analyses: pre-operative LOS rate ratio

Term	N	Rate Ratio	95% CI	p-value
Crude (unadjusted)	580	1.024	1.016–1.033	<0.001
Primary (adjusted)	580	1.032	1.021–1.042	<0.001
**S1: exclude post-op LOS >90 days**	578	1.029	1.02–1.039	<0.001
**S2: Elective patients**	318	0.976	0.923–1.037	0.3966
**S3: Urgent patients**	262	1.030	1.019–1.042	<0.001
**S4: CHH direct admission**	225	1.035	1.02–1.05	<0.001
**S5: Exclude in-hospital deaths (survival bias check)**	563	1.034	1.024–1.045	<0.001

**Table 4:** Fine-Gray competing risk model: time to discharge with in-hospital death as competing event

Variable	Sub-hazard ratio (SHR)	95% CI	*p*-value
**Pre-operative LOS (per day)**	0.915	0.899–0.931	< 0.001
**Age (per year)**	0.993	0.983–1.002	0.1322
**Valve only vs. CABG**	0.648	0.513–0.82	< 0.001
**CABG + Valve vs. CABG**	0.687	0.496–0.953	0.0246
**Major aortic vs. CABG**	0.345	0.242–0.492	< 0.001
**Other vs. CABG**	0.704	0.221–2.243	0.553

We considered the question ‘how does waiting time affect time-to discharge in a population that also faces a risk of in-hospital death?’ Fine-Gray competing risk model was used to analyse time to discharge with competing event being in-hospital death (Table 4). We adjusted for age and type of operation with SHR < 1 indicates cumulative probability of discharge is lower/slower discharge. Each additional pre-operative day reduces the sub distribution hazard of discharge alive by 8.5% (SHR 0.915, *p* < 0.001). This meant that the longer patients waited before surgery, the slower are discharged which is consistent with the negative binomial finding after accounting for competing risk of death. When compared to CABG, all other types of cardiac surgery led to longer post-operative stay with each additional pre-operative day stay. However, valve and major aortic surgery were statistically significant (*p* < 0.001) in prolonging post-operative stay. Patients undergoing isolated valve surgery had 35% lower sub distribution hazard of discharge compared with CABG patients (SHR 0.648, CI 0.513–0.82, *p* < 0.001). Patients undergoing major aortic surgery had 65.5% lower sub distribution hazard of discharge compared to CABG patients (SHR 0.345, 95% CI 0.242–0.492, *p* < 0.001) (Table 4).

**Table 5:** Urgency ratio with and without pre-operative LOS

Model	Pre-op LOS	Urgency RR (95% CI)	*p*-value
**Without pre-op LOS**	Excluded	1.2 (1.063–1.354)	0.003
**With pre-op LOS (full model)**	Included	0.904 (0.781–1.047)	0.174

We considered the impact of urgency of surgery on post-operative LOS. To do this, we analysed the urgency RR (comparing urgent to elective) with and without pre-op LOS. When pre-operative LOS was removed, urgency RR 1.2, 95% CI 1.063–1.0054, *p* = 0.003 indicating urgent patients stayed 1.2 times longer that elective patients. However, when the pre-operative LOS was included, the urgency RR was 0.904, 95% CI 0.781–1.047, *p* = 0.174. The impact of urgency on post-operative stay was overshadowed by the pre-operative LOS leading to the difference in RR.

## Discussion

Cardiac centres in the UK covers catchment areas of varying sizes. CHH serves an area of over a million hectares, with 8 DGH and a population of 1.7 million. Whilst patients could present directly to CHH, a significant proportion could be referred from DGH which provides different levels of services. Some of the DGH do not have out-of-hours cardiology services, PPCI or even cardiology services. CHH is a tertiary trauma teaching hospital with three cardiothoracic theatres, 12 bed cardiac intensive care, 6 cardiac surgeons and 8 cardiothoracic anaesthetist. Each theatre will aim perform two heart operations (graft(s), valve(s) or aortic surgery) per day. In some situations of high surgical complexity, (e.g.: aortic debranching) one cardiac case will be scheduled with additional smaller cases (e.g.: cardioversion). The ability to fully utilise theatres is linked to the availability of ICU beds which is affected by the availability of ward beds. Other issues that could impact theatre utility is theatre staffing which is affected by unpredictable out-of-hours or emergency work as there is no separate emergency theatre. This means in-hours emergency may lead to cancellation of a less urgent patient, and out of hours emergency work may lead to closure of theatres in the morning due to inadequate staffing.

Our evaluation of local service data from 2022 showed significant heterogeneity in patient waiting times for acute cardiac surgery; patients presenting directly to CHH waited a median of 12 days (IQR 8.0–15.6) for surgery, compared to those referred from DGH who waited a median of 5 days (IQR 2.0–9.2) before surgery. Our data does not capture the duration of pre-operative stay in the DGH whilst waiting for a bed in CHH. In some circumstances, patients waited > 50 days in CHH before receiving surgery. Whilst this does not reflect the majority, just over one-fifth of patients received surgery within a week of hospital arrival. Angiograms appeared to be conducted in a timely fashion for the majority of patients that were admitted with ischaemic heart disease. Patients could be in hospital for up to 11 days before being discussed by the MDT and then face further waiting time before surgery. There were multiple causes of this delay. There were instances where patients needed to be pre-optimised, receive treatment for infection, or required further specialist referral such as dental extraction, lack of intensive care beds, or lack of operating time due to unforeseen circumstances. However, we are unable to determine the reason(s) in all cases.

Post-operative LOS was affected by presenting EF, type of operation, age and duration of pre-operative stay. Poorer presenting EF, major aortic surgery, extreme of ages (below 40 and above 70 years old) and longer pre-operative stay impacted the post-operative stay. We were particularly interested in the impact of pre-operative LOS on post-operative LOS. Negative binomial regression analysis showed that urgent patients stayed 3% longer post-surgery per pre-operative day stay after adjusting for confounders. We are able to capture the full duration of hospital stay for those presenting to CHH directly. In this population RR was 1.035 (*p* < 0.001) per pre-operative day stay. We considered the impact of survival bias on post-operative LOS. We performed a sensitivity analysis excluding the 17 in hospital deaths; the RR 1.034 which is consistent with the primary estimate of RR 1.032 indicating that the deaths did not materially drive the result. The 17 patients that died after the surgery had their post-operative stay truncated. The Fine-Gray model used to address the issue of post-operative LOS truncation by treating death as a competing event rather than a censored observation in the time-to-discharge analysis demonstrated SHR 0.915. We considered the impact of urgency of surgery itself on post-operative LOS. Our study found that pre-operative LOS attenuated the impact of surgical urgency. When pre-operative LOS was removed from the adjusted model, operative urgency became a strong independent predictor of post-operative stay (RR 1.20, 95% CI 1.06–1.35, *p* = 0.003). Its attenuation to non-significance (RR 0.904, *p* = 0.174) upon re-inclusion of pre-operative LOS is consistent with complete mediation: the mechanism through which urgency prolongs recovery is the in-hospital wait itself, not the urgency classification. This reframes the clinical implication from patient selection to service delivery: reducing pre-operative delays in the urgent pathway is the actionable target.

### Limitations

Our data was unable to capture the full impact of the waiting times for patients on the acute cardiac pathway. Pre-operative LOS for patients transferred from DGH is left-censored at the point of arrival at the tertiary centre. The true exposure is longer than recorded and the estimated rate ratio for this sub-group should be regarded as conservative. Although patients from DGH were supposed to arrive ready for surgery, over 30% still waited for more than one week for surgery. We do not know the impact of waiting on mental and physical health, severity of symptoms or if anyone died whilst waiting for acute surgery.

This is a single-centre, observational analysis. Despite our best efforts, out of 585 patients, 5 had incomplete data and as such were excluded from analysis. Due to the small number of complications, the competing risk model is substantially underpowered for complications such as mortality, neurological, renal, and sternal wound infections.

CHH hospital was one of the most severely disrupted hospital in terms of the number of cardiac surgery performed in 2020; almost 50% reduction in activity [12]. We began the data analysis in 2022, although activity level has increased since then, NICOR data in 2025 reported that the activity level has not returned to pre-pandemic levels. The reduction in cardiac surgical activity was evident for all operations [13]. Our analysis captured the service at its low point, it remains to be seen if this is purely a post COVID impact. However, the services has not completely recovered 5 years post pandemic.

Another limitation is in the inability to perform a complete root cause analysis for the delays and identify any recurring theme that could be improved. Our data indicated potential reasons for waiting. However, it does not identify average waiting times for each factor. We are unable to trace the delays, investigations and challenges faced in the DGH setting. This could have been interesting as any investigations or interventions not completed pre-operatively in the DGH would translate to an extended waiting time in CHH. This would not only prolong their stay but impact the ability to optimise the capacity.

We considered the impact of survival bias on our study. As such, we performed sensitivity analysis. Two distinct mechanism were identified; firstly, pre-operative attrition: patients who died waiting for their surgery and never got to theatre were not captured in our data. Our data only includes those who survived to make it to theatre. Secondly, post-operative LOS truncation: 17 patients died during their post-operative stay. This meant that their post-operative LOS has been truncated. Both survival bias mechanism operate in the same direction: pre-operative attrition removes the sickest patients before surgery and in-hospital deaths (*n* = 17) truncated observed post-operative LOS. Each attenuates the estimated rate ratio implying the true association is at least as large as reported. Sensitivity analysis S4 which excludes death, addresses the second mechanism; the first cannot be tested with the available data.

## Conclusions

The longer urgent patients stay in hospital before their surgery, the longer their post-operative stay. Whilst a period of waiting maybe unavoidable or perhaps even necessary, our study findings support a service improvement focus on reducing pre-operative in-hospital delays for urgent cardiac surgery patients. Whether structured prehabilitation during the waiting period can further mitigate the effect warrants investigation in a study designed to test it, but the current data suggest the priority is getting patients to theatre faster, not adding a programme to the wait.

## Data Availability

No datasets were generated or analysed during the current study.

## Declarations

### AI tools

No AI-assisted writing tools were used in preparation of this manuscript.

## References

[1] Moscelli G, Siciliani L, Tonei V. Do waiting times affect health outcomes? Evidence from coronary bypass. Soc Sci Med. 2016;161:151–9. 10.1016/j.socscimed.2016.05.043. 27299977

[2] Sampalis J, Boukas S, Liberman M, Reid T, Dupuis G. Impact of waiting time on the quality of life of patients awaiting coronary artery bypass grafting. CMAJ. 2001;165(4):429–33. PMC81367 11531051

[3] National lnstitute for. Cardiovascular Outcomes Research (NICOR). 2022.

[4] National Institute for cardiovascular outcome research( < NICOR): 2020 summary report.London : NICOR> [Internet]. 2020

[5] Chen Y, Almirall-Sánchez A, Mockler D, Adrion E, Domínguez-Vivero C, Romero-Ortuño R. Hospital-associated deconditioning: not only physical, but also cognitive. Int J Geriatr Psychiatry. 2022. 10.1002/gps.5687. PMC9303382 35142397

[6] Fovet T, Guilhot C, Stevens L, Montel V, Delobel P, Roumanille R, et al. Early deconditioning of human skeletal muscles and the effects of a thigh cuff countermeasure. Int J Mol Sci. 2021. 10.3390/ijms222112064. PMC8584355 34769492

[7] National Institute for cardiovascular outcome research(<NICOR ): 2022 summary report. London.NICOR.

[8] Hulzebos EHJ, Helders PJM, Favié NJ, De Bie RA, Brutel de la Riviere A, Van Meeteren NLU. Preoperative intensive inspiratory muscle training to prevent postoperative pulmonary complications in high-risk patients undergoing CABG surgeryA randomized clinical trial. JAMA. 2006;296(15):1851–7. 10.1001/jama.296.15.1851. 17047215

[9] Sandhu M, Akowuah E. Does prehabilitation improve outcomes in cardiac surgical patients? Interact Cardiovasc Thorac Surg. 2019. 10.1093/icvts/ivz131. 31257409

[10] Gibbison B, Murphy GJ, Akowuah E, Loubani M, Pufulete M. Pre-operative and prehabilitation services in UK cardiac surgery centres. Anaesthesia. 2023;78(3):388–91. 10.1111/anae.15918. PMC10099756 36394827

[11] Bojar RM. Manual of perioperative care in adult cardiac surgery. Sixth edition. Hoboken, NJ: Wiley-Blackwell; 2020.

[12] National lnstitute for Cardiovascular Outcomes Research (NICOR) 2022 [Internet]. 2022. Available from: https://www.nicor.org.uk

[13] National Institute for Cardiovascular Outcomes research (NICOR) 2025 [Internet]. 2025. Available from: https://www.nicor.org.uk/~documents/route%3A/download/3663

